# Reply to Chirumbolo, “Antimicrobial regimens for the treatment of pan-drug-resistant *Acinetobacter baumannii* infections: some comments”

**DOI:** 10.1128/aac.00107-26

**Published:** 2026-04-30

**Authors:** Konstantinos Pontikis, Ilias Karaiskos, Helen Giamarellou, George L. Daikos

**Affiliations:** 1 Chest Diseases Hospital ‘I Sotiria’, Intensive Care Unit 1st Department of Pulmonology, National & Kapodistrian University of Athens 848121, Athens, Greece; 2 Hygeia General Hospital, 1st Department of Internal Medicine-Infectious Diseases, Athens, Greece; 3 2nd Department of Internal Medicine, Mitera General Hospital, Athens, Greece; Houston Methodist Hospital and Weill Cornell Medical College, Houston, Texas, USA

## REPLY

We thank S. Chirumbolo for his interest in the DESPAIR study and for the opportunity to clarify methodological and interpretative aspects of our work (1). Being the second of two independent commentaries, this critique underscores the clinical relevance of pan-drug-resistant *Acinetobacter baumannii* (PDR-AB) infections and the interest generated by this challenging therapeutic field.

The Author raises concerns regarding residual confounding, statistical power, and outcome interpretation, issues inherent to observational research. We fully agree that, as with all non-randomized studies, residual confounding cannot be completely excluded and that causal inference must therefore be made with appropriate caution. However, these limitations are generic to the study design and do not represent study-specific flaws nor undermine the internal consistency or robustness of our findings, which were explicitly acknowledged and addressed in the original DESPAIR publication (2).

We argue herein that the estimated size of the effect is large enough to be ignored. If this difference was due to confounding factors, these would have to be quite strongly associated with both treatment selection and clinical outcome and, at the same time, be absent from the extensive list of variables that we collected (as is evident from the tables in the Manuscript and its Supplement).

Another point that warrants emphasis is the direction of baseline imbalance. Patients receiving the meropenem-based regimen had markers of greater clinical severity at infection onset, including higher ICU admission rates, more frequent invasive mechanical ventilation, and higher prevalence of ventilator-associated pneumonia. If residual confounding were the dominant driver of the observed associations, it would be expected to bias results against this group. The observation of more favorable outcomes despite greater baseline severity is incompatible with confounding as the primary explanation. Instead, it is fully consistent with the enhancement of the treatment effect after adjustment using inverse probability of treatment weighting (IPTW). Indeed, we demonstrated through IPTW, which can be an efficient method even in small sample sizes (3, 4), increasing odds and hazard ratios for clinical failure and mortality, respectively, albeit with an unavoidable reduction in precision (2). Additionally, the extensive literature suggesting caution with tigecycline treatment in critically ill patients serves as a means of external validation of our results (5, 6).

Regarding the power of our study, no formal power analysis was performed, as is frequently the case with observational studies. We believe, however, that the discussion of power would be more relevant in the case of negative results and not in a study that readily shows differences in treatment effect.

The wide confidence intervals are an unavoidable consequence of our population size and an acknowledged limitation. Furthermore, it is not unusual for studies such as ours to exhibit imprecise estimates; however, the crucial clinical question is mainly whether an intervention is superior and not the extent of the superiority.

The chosen outcome is obviously not perfect, since some of its components are indeed prone to subjective evaluation; however, we believe that it describes pragmatically what physicians consider a failure. An analogous outcome was used in the OVERCOME randomized trial (7), thereby facilitating international comparisons. Regarding the validity of the results, we believe that the signal emitted by the difference in clinical failure rates is enhanced by aligning findings in secondary, and obviously more objective, outcomes of mortality and multi-organ dysfunction evolution. As mentioned by the Author, our effort to decrease subjectivity by performing a sensitivity analysis omitting the “withdrawal due to toxicity” criterion did not essentially alter our results. In contradiction to the Author, we consider verbal inclusion in the Manuscript and detailed provision of sensitivity analyses in the Supplement as transparent reporting.

As Professor Chirumbolo notes, having a central laboratory would increase the quality of our study; however, the logistic requirements were prohibitive for an unfunded study. Nevertheless, all participating laboratories have extensive experience with Acinetobacter infections, and we do not expect that the presence of a central laboratory would essentially alter our results. Broth microdilution was indeed not used in all cases, but it was used in more than 90% of them. It should be noted that compared to gold standard broth microdilution, alternate *in vitro* methods usually fail to detect resistance and not susceptibility (8). Regarding MIC reporting, as required per the standard nomenclature (9), all tested pathogens had MICs greater than 2 mg/L for colistin and tigecycline and greater than 8 mg/L for ampicillin-sulbactam.

We thank the author for the mathematical modeling of the implications of triple-regimen prescription on the epidemic of carbapenem resistance. However, we remain skeptical whether the preference of tigecycline over meropenem in addition to colistin-ampicillin/sulbactam (i.e., not following our results) in PDR-AB infections would lead to a meaningful reduction in the incidence of carbapenem-resistant infections.
